# Substance and alcohol use in pregnant women attending antenatal care at a tertiary hospital in Johannesburg, South Africa

**DOI:** 10.4102/sajpsychiatry.v31i0.2444

**Published:** 2025-05-14

**Authors:** Rebone I. Sebothoma, Sergius C. Onwukwe

**Affiliations:** 1 Department of Psychiatry, Faculty of Health Sciences, University of the Witwatersrand, Johannesburg, South Africa; 2 Department of Psychiatry, Life Brackenview Hospital, Johannesburg, South Africa; 3 Department of Family Medicine, Saint John Regional Hospital, Saint John, Canada; 4 Department of Family Medicine, Faculty of Health Sciences, University of the Witwatersrand, Johannesburg, South Africa

**Keywords:** substance use, pregnancy, prevalence, antenatal care, foetus, South Africa

## Abstract

**Background:**

Substance and alcohol use during pregnancy confers significant risk to the mother and foetus. Substance and alcohol use is common in South African general population. However, there is a paucity of literature on the extent of the problem among pregnant women.

**Aim:**

This study assessed the prevalence of substance use and its predictors among pregnant women attending antenatal care (ANC) at a tertiary hospital in Johannesburg, South Africa.

**Setting:**

This study was conducted at Rahima Moosa hospital, Johannesburg.

**Methods:**

This study was a retrospective record review of 399 consecutively selected pregnant women attending ANC. Socio-demographic, clinical, and substance use data were extracted and analysed using descriptive statistics and multivariate analyses.

**Results:**

Most pregnant women (84%) were aged between 20 years and 40 years. Substance use was documented in 45% (*N* = 178) of the records. Of these, concurrent use of alcohol and tobacco was 63% (*n* = 113). Factors that predicted the use of substances in pregnancy were low birth weight (aOR = 2.5, 95% CI = 1.23, 5.16, *p* = 0.01) and a positive HIV status (aOR = 0.6. 95% CI = 0.35, 0.96, *p* = 0.04).

**Conclusion:**

There was a high prevalence of substance use among pregnant women in the context of this study.

**Contribution:**

The increased risk of contracting HIV and having babies with low birth weights when substances are used in pregnancy highlights the need for appropriate behaviour modification for these women during antenatal care and this is in line with the health belief model.

## Introduction

The use of substances, especially during pregnancy, is a significant public health problem and this includes substances such as alcohol, tobacco, cannabis, amphetamine-type stimulants, cocaine, opioids, benzodiazepines, solvents, inhalers, and illicit and prescription drugs.^1,2,3^ These substances, when used in pregnancy, confer significant physical, clinical, social and psychological risks to both the mother and foetus.^1,2,3^ In terms of prevalence, there are well-documented variations in the use of these substances globally. However, evidence suggests that the most widely used substances in pregnancy are tobacco, alcohol, cannabis and illicit drugs.^4,5,6,7,8,9,10,11,12^ In South Africa, according to the reports of the Safe Passage Study (SPS), the prevalence of tobacco use in pregnancy is 56.3%, alcohol use is 61.2% and dual use of tobacco and alcohol in pregnancy is 37.4%.^13^ There is a paucity of data on the use of illicit substances in pregnancy in South Africa; however, in Cape Town, there are few reports on the use of illicit substances in pregnancy.^9,14^

The harm associated with maternal use and intrauterine exposure to alcohol, tobacco and illicit substances in pregnancy is well documented in the literature, extensive, and intergenerational.^5,7,9,10,15,16^ Specifically, smoking in pregnancy is not only associated with adverse pregnancy outcomes such as low birth weight, preterm birth and perinatal death. Still, it is linked to long-term maternal and foetal complications such as cardiovascular, respiratory, congenital, maxillofacial disease, cancers, and psychological, neurological and developmental problems.^4,5,15,16,17,18,19,20^

A common condition that has been widely reported in the literature that is associated with the use of alcohol in pregnancy is foetal alcohol spectrum disorder (FASD), a leading cause of non-inherited intellectual disability in the world, despite being 100% preventable through maternal abstinence from alcohol.^8,21,22^ The global prevalence of FASD is about 0.8%. Foetal alcohol syndrome (FAS), which is the most serious of FASDs, is known to cause alcohol-related birth defects and alcohol-related neurodevelopment disorders such as congenital malformations, intrauterine growth restrictions, intellectual disability, behavioural disorders, speech and language difficulties, visual and audiological impairments, cardiac deformities and urogenital problems.^1,8,21,22^ Moderate to heavy alcohol drinking during pregnancy is associated with deleterious and adverse pregnancy outcomes such as spontaneous miscarriage, stillbirth, low birth weight, intrauterine growth restriction, preterm delivery and infant mortality.^8,9,22^ Regrettably, South Africa has been reported to have the highest rates of FASD in the world, with FASD rates of 310 per 1000 documented in the Western Cape,^23^ and this underscores why the use of alcohol in pregnancy needs urgent public health intervention, especially as there is no safe dose in pregnancy.^5,8,24,25^

Cannabis, the most reported illicit drug used in pregnancy, and others such as cocaine, nonprescription opioids, and methamphetamine have been linked with preterm labour, low birth weight, anencephaly (a condition in which the forebrain fails to form), and later development of attention deficit hyperactivity disorder, learning disabilities, memory impairment, depression and aggression.^1,10,26^

Despite widespread public knowledge and awareness of its deleterious health effects, substance use in pregnancy continues and has been implicated in the development of maternal psychological, behavioural and socio-economic problems such as anxiety disorders or depression, problematic relationships, risky health behaviours, maladaptive functioning, low educational levels, single parenthood and low-income levels.^27,28^ Furthermore, according to published studies, some of the factors that may be associated with substance use in pregnancy include young maternal age, low educational level, poverty or unemployment, use of substances before pregnancy and interpersonal conflicts.^1,27,28^

In terms of screening for substance use in pregnancy, it is known that the use of substances in pregnancy is associated with preventable deleterious maternal and foetal outcomes, and this has necessitated the recommendation for the routine screening of all pregnant women as the standard of care.^29,30,31^ To underscore this, the World Health Organization (WHO) has published an evidence-based guideline regarding the identification and management of substance use in pregnancy.^3^ The screening methods include urinalysis, self-report questionnaires, hair analysis, urine and blood tests, meconium tests, nail analysis, self-report questionnaires such as ASSIST (Alcohol, Smoking, and Substance Involvement Screening Test) and AUDIT (Alcohol Use Disorders Identification Test), and each has its limitations.^3,29,30,31^ For example, compared to AUDIT, ASSIST is preferred for routine screening of substances because, unlike AUDIT, which screens for only alcohol use, it screens for all substances.^29,30,31^

Substance use in pregnancy is a health-compromising, risky, and behavioural problem. Therefore, designing an effective intervention may require using the individual behavioural theoretical framework, and the health belief model (HBM) offers this opportunity.^32,33,34^ In practice, the HBM is a historical, behavioural and theoretical framework that can be used to explain why people take actions or refuse to take actions against certain individual health risk behaviours such as substance use in pregnancy and in the design of interventions for such behaviours.^32,33,34^ It has been used in research to explain people’s health prevention, promotion and illness behaviours and in designing interventions.^32,33,34^ The HBM constructs are perceived susceptibility, severity, benefits, barriers, cues to actions and self-efficacy.^32,33,34^

Studies have been done on the use of substances in pregnancy globally, and data from most of these studies may not be adequate in highlighting the extent of the problem in other contexts that are different from where these studies were done.^4,5,6,7,8,9,10,11,12^ This is because substance use has important behavioural components that may differ among socio-demographic groups.^4,5,6,7,8,9,10,11,12^ Specifically, in South Africa, most of these studies have been done in the Western Cape, and the fact that substance use in pregnancy is still persistent highlights why additional data from other population groups in South Africa is essential.^4,13,14,15^ To the best of the author’s knowledge, there is a paucity of data on substance use in pregnancy in the context of this study in Gauteng, South Africa. Therefore, this study aims to assess the prevalence of substance use and its predictors among pregnant women accessing antenatal care at a tertiary hospital in Johannesburg, South Africa. It is hoped that the findings of this study will provide additional insight into the use of substances in pregnancy in South Africa and into the design of interventions for it in line with the objectives of the HBM.

## Research methods and design

### Study design and setting

This was a cross-sectional retrospective review of maternity or antenatal case records of pregnant women who attended antenatal care (ANC) and delivered at Rahima Moosa Mother and Child Hospital from the first to the third week in January 2013. This hospital is situated in Coronation Ville, Johannesburg, Gauteng, South Africa, and provides inpatient and outpatient tertiary-level obstetric and paediatric services to the inhabitants of that area. Furthermore, the hospital is a clinical training site for medical students, interns, and the University of the Witwatersrand registrars. Data collection was from April 2014 to September 2014. It is acknowledged that there may be a minor overlap of information in this article with a previous report on this topic used for postgraduate study.^35^ However, the focus of what is discussed in this report is different.

### Study population, sample size, and sampling methods

The study included all the socio-demographic and clinical information documented in the maternity or antenatal case records of pregnant women, irrespective of age, who delivered at Rahima Moosa Hospital, between 01 January 2013 and 21 January 2013. According to their records, approximately 970 pregnant women delivered their babies at this hospital in January 2013. Therefore, assuming a margin of sample error of 5%, a confidence level of 95%, and a response distribution of 50%, a sample size of 399 pregnant women was found to be adequate, and these maternity case records or antenatal cards were consecutively selected for the study.^36^ The hospital numbers of all the pregnant women who had their deliveries during the study period were obtained from the maternity admissions book. They were taken to the hospital records office for extraction of the maternity or antenatal case records. At the end of each day of the data collection, the patients’ maternity or case records were marked with a sticker to avoid repeat selection, which continued until the required sample size was reached.

### Data collection

Data were collected manually from the pregnant women’s maternity or antenatal case records and entered on an Excel^®^ data collection sheet. This maternity or antenatal case record is the recommended data collection and documentation tool for all pregnant women in South African public health institutions. Specifically, the maternity or antenatal case records have sections that collect data for both the dependent and independent variables used for this study. Usually, these data are collected routinely as a standard practice by self-reporting during medical and general history taking when a pregnant woman enrols for antenatal care in any South African public health institution designed for this purpose. These data are also updated regularly throughout the pregnancy. Therefore, for this study and based on the documentation on the maternity or antenatal records at the study site, information on socio-demographic data (age, race, relationship status, employment status and level of education), clinical data (gestational age at delivery, parity, booking status, human immunodeficiency virus [HIV] status, method of delivery, birth weight of baby and follow-up with psychological or psychiatric services), and substance use data (alcohol, tobacco, cannabis, other, unknown) were collected and subsequently used for data analysis.

### Data analysis

Data were analysed using STATA^®^ version 13.0 (StataCorp LLC [Limited Liability Company] College Station, Texas, United States). All descriptive statistics were done using categorical data and presented as percentages and frequencies. The age of the pregnant women, which is continuous data, was also presented as a median. For inferential statistics, a preliminary test of association between independent variables and substance use in pregnancy was done using the Chi-square test. This is justified because it is the initial test for statistical significance between two categorical variables.^37,38,39^ Statistically significant variables obtained from the bivariate analysis were entered into a model for multivariate regression analysis, and this determined the socio-demographic and/or clinical factors that were associated with substance use in pregnancy. The justification for using stepwise multivariate regression analysis in this study is that it is the gold standard statistical test when there is a need to analyse the influence of multiple independent variables on a dependent variable, which in this study is substance use in pregnancy.^37,38,39^ Additionally, multivariate regression controls and adjusts confounding variables for a true association between the independent and dependent variables to emerge.^37,38,39^ A significant probability level was set at a *p*-value less than 0.05.

### Ethical considerations

Permission for the study was obtained from the Chief Executive Officer of Rahima Moosa Mother and Child Hospital. Ethical approval was obtained from the Human Research Ethics Committee of the University of the Witwatersrand, South Africa (Certificate number: M131188). All identifying data in the reviewed files, including patients’ names, were coded to maintain confidentiality.

## Results

**FIGURE 1:**
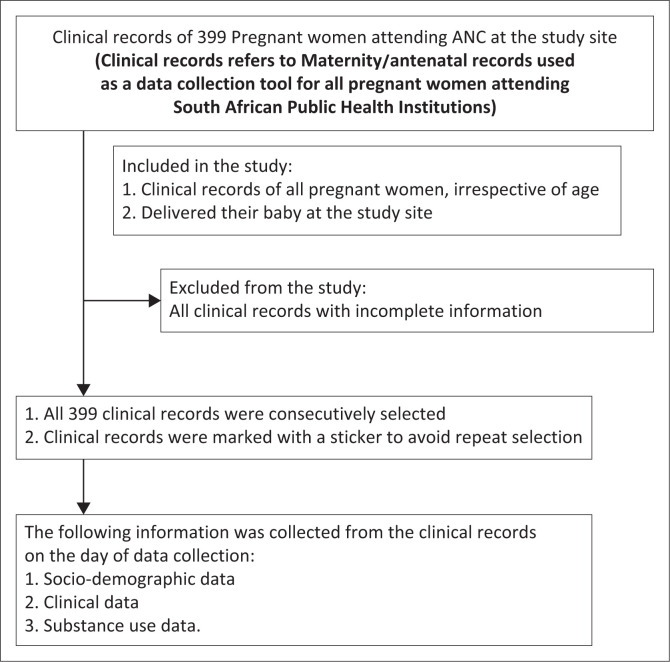
Flow diagram of study sample.

Figure 1 illustrates the flow diagram of the study sample.

### Socio-demographic characteristics

**FIGURE 2:**
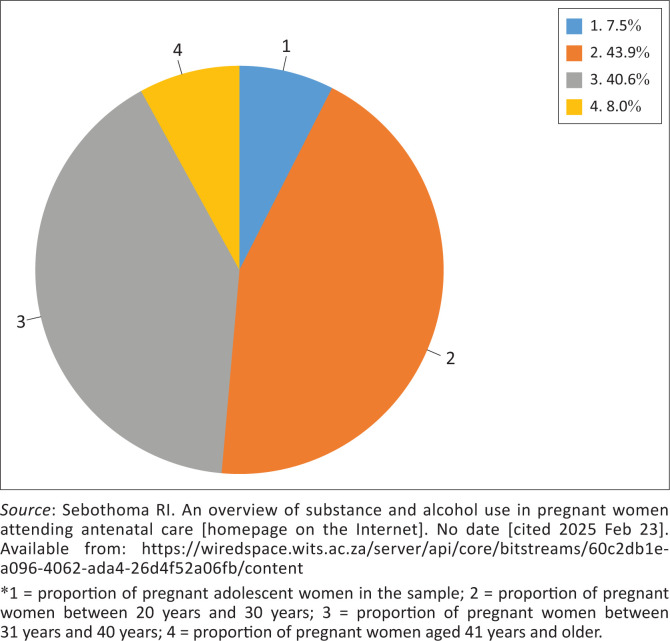
Distribution of sample by age group.

Pregnant women who were aged between 20 years and 30 years, 43.9% (*n* = 175) with a median age of 25 years, and those who were aged between 31 years and 40 years, 40.6% (*n* = 162) with a median age of 36 years were in the majority, 84.5% (*n* = 337). Adolescents with a median age of 16 years accounted for 7.5% (*n* = 30) of the sample, and pregnant women aged 41 years and older accounted for 8% (*n* = 32). The details of the distribution of the sample by age group are shown in Figure 2.^40^

As shown in Table 1,^35,40^ in terms of race, the majority of the pregnant women, that is, 79% (*n* = 316) were black African, 16% (*n* = 64) were coloured, 3.8% (*n* = 15) were Indian or Asian, and 1% (*n* = 4) were white. Almost two-thirds, 61%, (*n* = 243) were single, 33% (*n* = 131) were married and 6% (*n* = 25) were either separated, divorced, or had no information on marital status. As regards employment, over half (52%, *n* = 207) of the pregnant women in this study were unemployed. There was no information on the level of education in almost all the files or clinical records used for this study.

**TABLE 1:** Socio-demographic characteristics (*N* = 399).

Variables	Categories	Frequency	%
Population group†	Black African people	316	79
	Coloured people	64	16
	Indian or Asian people	15	4
	White people	4	1
Marital status	Single	243	61
	Married	131	33
	Separated or widow or other	25	6
Employment status	Unemployed	207	52
	Employed	131	33
	Unknown	61	15
Highest level of education	Completed grade 12	1	1
	Unknown	398	99

*Source*: Sebothoma RI. An overview of substance and alcohol use in pregnant women attending antenatal care [homepage on the Internet]. No date [cited 2025 Feb 23]. Available from: https://wiredspace.wits.ac.za/server/api/core/bitstreams/60c2db1e-a096-4062-ada4-26d4f52a06fb/content; Statistics South Africa. Mid-year population estimates [homepage on the Internet]. 2011 [cited n.d.]. Available from: https://www.statssa.gov.za/publications/P0302/P03022011.pdf

Note: Statistics South Africa uses this nomenclature to classify the different population groups in South AfricaXXX (Statistics South Africa, 2011).

† , Population group = black African, coloured, Indian/Asian and white.

### Clinical characteristics

The clinical characteristics of the pregnant women are shown in Table 2.^35^ Majority of the women (88%, *n* = 351) delivered their baby at full term by caesarean section (64%, *n* = 25), and had 1–2 children (71.7%, *n* = 286). Incidentally, 21% (*n* = 82) of the pregnant women were HIV positive, and a small proportion gave birth to children with low birth weight (10%, *n* = 40). Almost all the pregnant women in this study were booked for antenatal care (99.7%, *n* = 398), and none were recorded to have ever had contact with psychological or psychiatric services.

**TABLE 2:** Clinical characteristics (*N* = 399).

Variables	Categories	Frequency	%
Gestation at delivery	Full term	351	88.0
	Premature	47	11.8
	Unknown	1	0.2
HIV status	Negative	312	78.0
	Positive	82	21.0
	Unknown	5	1.0
Parity	1–2	286	71.7
	3–5	112	28.0
	> 5	1	0.3
Method of delivery	Vaginal	143	36.0
	Caesarean	256	64.0
Birth weight	Normal	359	90.0
	Low	40	10.0
Antenatal care booking	Booked	398	99.7
	Not booked	1	0.3
Mental health services	No	399	100.0
	Yes	0	0.0

*Source:* Sebothoma RI. An overview of substance and alcohol use in pregnant women attending antenatal care [homepage on the Internet]. No date [cited 2025 Feb 23]. Available from: https://wiredspace.wits.ac.za/server/api/core/bitstreams/60c2db1e-a096-4062-ada4-26d4f52a06fb/content

Note: Parity = number of completed viable pregnancies in a substance user.

HIV, human immunodeficiency virus.

### Prevalence, types, and characteristics of substance use

Of the 399 pregnant women whose maternity case records or clinical records were used for this study, 45% (*n* = 178) were documented as using substances. No record of substance use was found in 55% (*n* = 221). Tobacco use was documented in 37% (*n* = 149) of the clinical records, alcohol use in 35% (*n* = 138) and cannabis in 1% (*n* = 4). The use of illicit substances was not documented in any patient files/clinical records.

**FIGURE 3:**
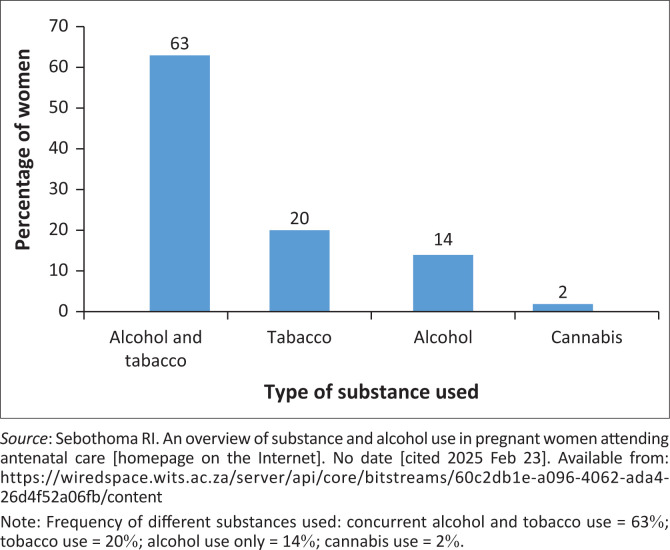
Frequency of substances used in pregnancy.

As shown in Figure 3,^35^ of the 178 clinical records in which substance use was documented, 63% (*n* = 113) of the pregnant women used both alcohol and tobacco, 20% (*n* = 36) used only tobacco, 14% (*n* = 25) used alcohol, and 2% (*n* = 4) used cannabis.

### Determinants of substance use in pregnancy

Initial bivariate analysis using the Chi-square test indicated that none of the socio-demographic variables were significantly associated with substance use.

However, as shown in Table 3,^35^ there were significant associations between substance use and gestational age (preterm) delivery (*p* < 0.0001), HIV status (*p* = 0.04) and birth weight (*p* = 0.001) in bivariate analysis.

**TABLE 3:** Association between substance use and clinical factors.

Variables	No substance use		Substance use		*p*-value
	*n*	%	*n*	%	
**Delivery gestation**	-	-	-	-	< 0.0001*
Full term	212	60	139	40	-
Premature	13	28	34	72	-
**HIV status**					
Negative	185	59	127	41	0.04*
Positive	38	46	44	54	-
**Parity**	-	-	-	-	0.16
1–2	155	54	131	46	-
≥ 3	70	62	43	38	-
**Method of delivery**	-	-	-	-	0.48
Vaginal	84	59	59	41	-
Caesarean	141	55	115	45	-
**Birth weight**	-	-	-	-	0.001*
Normal	212	59	147	41	-
Low	13	32	27	68	-

*Source:* Sebothoma RI. An overview of substance and alcohol use in pregnant women attending antenatal care [homepage on the Internet]. No date [cited 2025 Feb 23]. Available from: https://wiredspace.wits.ac.za/server/api/core/bitstreams/60c2db1e-a096-4062-ada4-26d4f52a06fb/content

Note: Number of non-substance users in pregnancy (*n*) = (55% or 221); number of substance users in pregnancy (*n*) = (45% or 178); parity = number of completed viable pregnancies in a substance user.

* , *p*-value is set at less than 0.05.

These significant variables were then included in a stepwise multivariate logistic regression analysis model to determine which factors predicted substance use in pregnancy (Table 4).^35,40^ This also ensures that the confounding variables are effectively removed or adjusted. In this study, preterm delivery was initially significant in the bivariate analysis but disappeared in the multivariate regression, so it is most likely a confounder.^37,38,39^ The results indicate substantial associations of substance use with HIV status and birth weight. Specifically, women who were HIV negative were less likely to use substances (adjusted odds ratio [aOR] = 0.6, 95% confidence interval [CI] 0.35–0.96, *p* = 0.04) than women who were HIV positive. Additionally, women using substances were 2.5 times more likely to have a child with low birth weight than non-users (aOR = 2.5, 95% CI: 1.23–5.16, *p* = 0.01).

**TABLE 4:** Multivariate analysis of correlates of substance use.

Substance use correlate	AOR	95% CI	Baseline	*p*
**Population group**	-	-	-	0.64
Coloured	-	-	1 [Reference]	-
Black	1.1	0.65–2.00	-	-
**HIV status**	-	-	-	0.04*
Positive	-	-	1 [Reference]	-
Negative	0.6	0.35–0.96	-	-
**Parity**	-	-	-	0.18
1–2	-	-	1 [Reference]	-
≥ 3	0.7	0.45–1.16	-	-
**Birth weight**	-	-	-	0.01*
Normal	-	-	1 [Reference]	-
Low	2.5	1.23–5.16	-	-

*Source:* Sebothoma RI. An overview of substance and alcohol use in pregnant women attending antenatal care [homepage on the Internet]. No date [cited 2025 Feb 23]. Available from: https://wiredspace.wits.ac.za/server/api/core/bitstreams/60c2db1e-a096-4062-ada4-26d4f52a06fb/content; Statistics South Africa. Mid-year population estimates [homepage on the Internet]. 2011 [cited n.d.]. Available from: https://www.statssa.gov.za/publications/P0302/P03022011.pdf

CI, comparison index; HIV, human immunodeficiency virus; AOR, adjusted odds ratio; CI, confidence interval.

* , Significant *p*-value is set at less than 0.05.

## Discussion

In this study, 45% of the pregnant women who delivered their babies at Rahima Moosa Mother and Child Hospital were found to have used substances. Specifically, 37% used tobacco, 35% used alcohol, 1% used cannabis and 63% used tobacco and alcohol concurrently. There was no record of the use of illicit substances among the study samples. These findings are concerning because the deleterious outcomes associated with substance use in pregnancy are well reported in the literature.^4,5,15,16,17,18,19,20^ and become even more worrisome because there is evidence suggesting that South Africa has the highest prevalence of FASD and FAS in the world.^23^ Coincidentally, in this study, over one-third of the pregnant women used alcohol, the single risk factor that has been implicated in the pathogenesis of FASD and FAS.^8,21,22,23^ It is important to emphasise that our finding on the prevalence of substance use in pregnancy is higher than most international reports.^5,6,7,8,11,15,22,27,41,42,43^ However, it is in line with the global reports on the relative use of each of these substances in terms of frequency, where tobacco is said to be more widely used, followed by alcohol, cannabis and illicit drugs.^4,5,6,7,8,9,10,11,12^ Incidentally, in South Africa, there are mixed reports on the relative prevalence of each of these substances used in pregnancy. For example, according to the reports of the SPS, the prevalence of tobacco use in pregnancy is 56.3%, alcohol use is 61.2% and dual use of both tobacco and alcohol in pregnancy is 37.4%.^13^ This is markedly different from our findings and other reports on substance use in South Africa.^4,10,12,14,18,23^ This is not surprising because apart from the possible effects of the methodological differences used in these studies, there are variations in the use of these substances in pregnancy in terms of demography, cultural issues and individual behaviour.^4,10,12,14,18,23^ This notwithstanding, the level of substance use found in this study may even be an underestimate because a study done by Raggio et al.^44^ in South Africa and Uganda found that 16% of HIV-positive pregnant women under-reported their alcohol use in pregnancy when compared with the results of their biochemical analysis. Incidentally, in this study, 21% of the samples were HIV positive. Our findings that suggest that there were no records of the use of illicit drugs among pregnant women may be an underestimate because evidence shows that there is a global use of illicit substances in pregnancy, and this is associated with deleterious pregnancy outcomes.^4,5,6,7,8,9,10,11,12^ In our study, this may be due to under-reporting by pregnant women. According to McMillin et al.,^26^ under-reporting of the use of illicit drugs in pregnancy is common because women indulging in this behaviour generally have a fear of stigmatisation and possible legal consequences of disclosure of the use of illicit drugs. However, it is essential to emphasise that biochemical testing of urine or blood samples is the most objective and valid method of accurately assessing substances in pregnancy, as documented in the literature.^29,30,31^ We were unable to use this method to verify the reported substance use in pregnancy in the clinical records because of the design of the study, which is a retrospective record review. Therefore, our reported level of prevalence of substance use in pregnancy needs to be interpreted with caution because it may have been underestimated.

In our study, black Africans (79%), single mothers (61%) and those who are unemployed (52%) or whose education level is unknown are the socio-demographic groups with the highest frequency of use of substances during pregnancy. Although these variables did not achieve statistical significance in our study, they have important public health implications. However, we do not have data on how socio-demographic conditions affect pregnant women using substances. However, in a study done by Ünver and Alkan^45^ to explore how socio-demographic issues influence the use of substances among women in the general population, they found that those women who were younger, single, unemployed, had a higher education level, had support structures are the ones at increased risk of substance use, and should be prioritised for substance prevention, cessation and rehabilitation programmes.^45^ In our study, the majority of black Africans, who incidentally are the historically disadvantaged population in South Africa, are found to be using substances more disproportionally than other racial groups in the study. They are also the population groups that are at increased risk of poor maternal health outcomes, socio-economic determinants of health and psycho-social stressors.^4,5,15,16,17,18,19,20^ These, no doubt, are important risk factors for substance use in pregnancy.^4,5,15,16,17,18,19,20^ Furthermore, all the deleterious clinical outcomes reported in the literature due to substance use in pregnancy are more accentuated among the African black populations who are also less educated with a high level of unemployment.^4,5,15,16,17,18,19,20^ In a study done on a North American sample, the minority black population, who are also the group that is worst affected by unemployment, poverty, poor education and other factors that perpetuate social determinants of health, were found to be at increased risk of use of substances in pregnancy.^27^ This highlights why the opportunities offered by antenatal clinical encounters with health care providers should be maximised to screen, prevent and optimally care for this population group with potential socio-demographic and social determinants of health conditions that may increase their risk of use of substances in pregnancy.^1,27,28,45^

This study found that the use of substances in pregnancy is significantly associated with being HIV positive. Our finding is in line with previous studies,^46,47^ and in addition, some of these studies found an association with HIV vertical transmission to the baby.^47^ It is important to note that the association between substance use and HIV positivity is not surprising because this association has been well-established and described in the literature.^48^ In terms of what is known on this subject, substances, including alcohol, appear to increase the risk of HIV in so many ways, such as influencing individual behaviour, especially risky sexual conduct.^46,48^ Also, some potential biochemical effects of alcohol may compromise the immune system and liver function, where some of the substances are metabolised, thereby increasing the risk of acquiring HIV.^49^ Therefore, it may be helpful for providers of care to consider using the HBM when discussing risk perceptions and susceptibility to the use of substances in pregnancy during ANC visits and other clinical encounters.^32,33,34^

Another important finding of this study is that substance use is associated with low birth weight in infants. Specifically, we found that women using substances were 2.5 times more likely to have a child with low birth weight than non-users (aOR = 2.5, *p* = 0.01), and this is consistent with the report of several studies.^50,51,52,53,54^ Although the initial association we found in a bivariate analysis between substance use in pregnancy and preterm delivery disappeared in the multivariate regression analysis, possibly due to a confounding or smaller sample size effect, preterm delivery, which is related to low birth weight, is a well-described clinical outcome of tobacco, alcohol, cannabis and other illicit substance use in pregnancy.^1,5,8,9,10,15,16,22,26,28^ To underscore this, studies with large sample sizes have demonstrated strong associations between low birth weight/preterm delivery and use of substances in pregnancy.^50,51,52,53^

A substantial number of women who used substances during pregnancy in this study were teenagers/adolescents (7.5%; *n* = 30), and when extrapolated to the population level, it has important implications for public health. Although we did not find any association between socio-demographic variables and substance use in pregnancy, in one study, teenage pregnancy was found to be associated with substance use and poor socio-economic status, thus, highlighting its huge public health implications.^55^ It is well known that adolescent pregnancy alone may be associated with deleterious clinical, psychological and socio-economic outcomes, and when complicated by substance use, becomes more challenging for the mother, infant and health care system.^56,57^ Specifically, pregnant teenagers using substances are at an increased risk of pregnancy-related maternal and foetal outcomes such as obstructed labour, teenage-related sexually transmitted infections, maternal death, extremely low birth weight, sudden infant death syndrome, developmental delay and long-term substance withdrawal complications.^56,57^ This has massive implications for public health and the entire healthcare system because of the increased economic strain it puts on the scarce healthcare budget in delivering needed care to them.^55,56,57^

From a public health perspective, it is, therefore, essential that strategies are put in place to target this unique population of pregnant mothers even before they get pregnant. Extensive education and campaigns on the dangers of teenage pregnancy need to be prioritised as a prevention approach in schools, places of religious worship and communities.^55,56,57^ For those who are already pregnant, comprehensive prenatal care and antenatal care need to be optimised, and health care providers who care for them need to be empowered and resourced in the care of teenage pregnant mothers.^55,56,57^ Additionally, integrated screening and counselling for substance use and provision of addiction treatment harm reduction resources need to be part of the care plan for teenagers using substances during pregnancy.^55,56,57^ Also, in the long term, addressing the conditions that perpetuate social determinants of health that contribute to substance use by relevant governmental and nongovernmental agencies and policymakers would help reduce the socio-economic drivers of substance use in pregnancy.^24,28,55,56,57^ Encouraging family members, relatives, friends and the community on the need for support and care would go a long way in assisting them deal with the psychological problems and stigma associated with substance use during pregnancy.^24,25,55,56,57^ This fact needs to be remembered when designing interventions for teenagers who are pregnant and are also using substances.

### Strengths and limitations

This retrospective cross-sectional study involved a review of patient records selected consecutively and may be limited by misclassification of data, inadequate data, selection and information bias. Specifically, there was no record of documentation of the use of illicit drugs among pregnant women, which is due to under-reporting.^26^ The implication of this is that substance use in pregnant women may have been under-reported because the data that were extracted from the maternity case or antenatal records for this study were based on self-reports, which is inherent with inadequate documentation. Data used for analysis in this study were collected in 2014 and, although important, may be seen as old data. However, according to Näher et al.,^59^ it may be considered secondary data because the antenatal care providers collected it to care for pregnant women and not primarily for research. Also, this study found that low birth weight and a positive HIV infection were significantly associated with the use of substances in pregnancy. However, while this finding is biologically plausible and well described in the literature,^46,47,48,50,51,52,53,54^ for our study, which is a retrospective cross-sectional design, it is limited in evidence because the relationship cannot establish or infer causality.^37,38^ A longitudinal study design will assist in exploring this further.^37,38^ Rahima Moosa Mother and Child Hospital is a single-site tertiary hospital and receives referrals of pregnant women with a certain degree of obstetric risk from primary health care centres, so the result of this study may not be generalised to the entire population of pregnant women assessing care in the study site or beyond. Therefore, a future study with a larger sample from different sites that attend to antenatal women needs to be done to improve the generalisation of the findings from this critical public health topic. Notwithstanding these potential limitations, our study provides an estimate and additional information on the use of substances in pregnancy in a tertiary hospital setting. As suggested, it may offer opportunities for further studies in this subject.

## Conclusion

This study found that there was a high prevalence of substance use among pregnant women, and this has important implications for public health. The increased risk of contracting HIV and having babies with low birth weights and who may potentially have FASD or FAS when substances are used in pregnancy highlights the need for interventions to be started earlier, even before pregnancy, for better outcomes. Those missed should be targeted at their first antenatal visit. Specifically, because substance use is behavioural and modifiable, ongoing screening, primary prevention, counselling, health education and promotion and appropriate behaviour change intervention need to be the standard of care for all pregnant women.^24,25,28,58^ Thus, the HBM may be for risk reduction intervention in this study because almost half of the pregnant women used substances. This suggests that they possibly had poor risk perception of their susceptibility to adverse outcomes of the use of substances in pregnancy or how severe these outcomes would be. This is in line with the benefit of using the HBM, as we suggest in this study, to screen all pregnant women for this risk of substance use whenever they attend antenatal care.^32,33,34^ The practical importance of this is that the healthcare provider attending to these women can maximise every opportunity offered by their antenatal visits to use the HBM to explore and discuss their beliefs about the potential harm of the use of substances to their baby (perceived severity), their likelihood of experiencing deleterious consequences (perceived susceptibility), the benefits of stopping substance use (perceived benefits) and their confidence in their ability to stop using substances (self-efficacy).^32,33,34^ All these, when done correctly in a patient-centred manner without prejudice or being judgemental, have been shown to influence people’s decision to abstain from risky behaviour that has potentially devastating consequences for health and well-being. This, no doubt, applies to women using substances in pregnancy.^32,33,34^ Our suggestion for the use of the HBM, which is also evidence-based, may be helpful not only in designing interventions as described, but in understanding women’s behavioural risk perceptions for the use of substances in pregnancy,^32,33,34^ which has been clearly shown to be dangerous to their health and that of their baby.^56,57^

### Recommendations

The findings of this study indicate that risk stratification, screening, counselling, health education and promotion and behaviour modification for substance use should be the pivot of interventions for pregnant women accessing ANC services. The evidence-based health belief model should be the cornerstone for these interventions, which should be ongoing because of the identified risks and health consequences associated with substance use in pregnancy. Additional consideration should be given to teenagers who were found substantially to be using substances in pregnancy in this study. Healthcare professionals who provide care to pregnant women using substances should aim to understand their risk perception and why they are indulging in risky behaviour in line with HBM and patient-centredness. Specifically, antenatal care providers should adopt the WHO’s recommendation for the routine screening of all pregnant women for substance use as a primary prevention standard of care.^30,31^ Compared to AUDIT, antenatal care providers should use self-report screening questionnaires such as ASSIST, which can assess different substances, as a screening tool.^30,31^ Health education and counselling for at-risk pregnant women should be ongoing, and referrals should be made appropriately for those needing long-term specialised rehabilitation interventions.^30,31^ Collaboration needs to be made between all categories of care providers, relevant stakeholders and policymakers for the comprehensive and integrated prenatal substance use care programme for all pregnant women at risk of this avoidable public health problem. All of this would put the antenatal care provider in a better position to assist pregnant women using substances in overcoming their risky behaviour, and this may translate to a positive social change for this population. Although our study was not designed to assess why pregnant women use substances during pregnancy, there is a consideration of additional research to understand why they indulge in this risky behaviour that is causing harm to their health.
